# Lutein accumulates in subcellular membranes of brain regions in adult rhesus macaques: Relationship to DHA oxidation products

**DOI:** 10.1371/journal.pone.0186767

**Published:** 2017-10-19

**Authors:** Emily S. Mohn, John W. Erdman, Matthew J. Kuchan, Martha Neuringer, Elizabeth J. Johnson

**Affiliations:** 1 Jean Mayer US Department of Agriculture Human Nutrition Research Center on Aging, Tufts University, Boston, Massachusetts, United States of America; 2 Department of Food Science and Human Nutrition, University of Illinois at Urbana-Champaign, Urbana, Illinois, United States of America; 3 Discovery Research, Abbott Nutrition, Columbus, Ohio, United States of America; 4 Oregon National Primate Research Center, Oregon Health & Science University, Beaverton, Oregon, United States of America; Tokai University, JAPAN

## Abstract

**Objectives:**

Lutein, a carotenoid with anti-oxidant functions, preferentially accumulates in primate brain and is positively related to cognition in humans. Docosahexaenoic acid (DHA), an omega-3 polyunsaturated fatty acid (PUFA), is also beneficial for cognition, but is susceptible to oxidation. The present study characterized the membrane distribution of lutein in brain regions important for different domains of cognitive function and determined whether membrane lutein was associated with brain PUFA oxidation.

**Methods:**

Adult rhesus monkeys were fed a stock diet (~2 mg/day lutein or ~0.5 μmol/kg body weight/day) (n = 9) or the stock diet plus a daily supplement of lutein (~4.5 mg/day or~1 μmol/kg body weight/day) and zeaxanthin (~0.5 mg/day or 0.1 μmol/kg body weight/day) for 6–12 months (n = 4). Nuclear, myelin, mitochondrial, and neuronal plasma membranes were isolated using a Ficoll density gradient from prefrontal cortex (PFC), cerebellum (CER), striatum (ST), and hippocampus (HC). Carotenoids, PUFAs, and PUFA oxidation products were measured using HPLC, GC, and LC-GC/MS, respectively.

**Results:**

All-*trans*-lutein (ng/mg protein) was detected in all regions and membranes and was highly variable among monkeys. Lutein/zeaxanthin supplementation significantly increased total concentrations of lutein in serum, PFC and CER, as well as lutein in mitochondrial membranes and total DHA concentrations in PFC only (P<0.05). In PFC and ST, mitochondrial lutein was inversely related to DHA oxidation products, but not those from arachidonic acid (P <0.05).

**Discussion:**

This study provides novel data on subcellular lutein accumulation and its relationship to DHA oxidation in primate brain. These findings support the hypothesis that lutein may be associated with antioxidant functions in the brain.

## Introduction

Lutein, a dietary carotenoid found in spinach, kale, eggs, and corn [1], is known to selectively accumulate in the retina of primates where, along with its isomer, zeaxanthin, it forms macular pigment (MP) [2]. MP protects the eye from harmful blue light by acting as a filter. Lutein and zeaxanthin also serve as antioxidants to protect polyunsaturated fatty acids (PUFAs) in retinal photoreceptors from oxidation [3,4]. MP density is positively correlated to brain lutein concentrations in matched retina and brain tissue of humans and non-human primates [5,6]. Studies in both infants and adults have shown that, despite not being the predominant carotenoid in the diet, concentrations of lutein in the human brain are higher than those of other carotenoids [7,8]. Preferential accumulation of lutein in the brain during multiple life stages may be indicative of an important function.

Accumulating evidence demonstrates that serum and brain lutein concentrations, as well as MP density, are positively related to cognitive function in older adults and centenarians [8–11]. Several intervention studies have also reported a beneficial effect of lutein on cognitive performance. Consumption of avocados, a highly bioavailable source of lutein [12], for 6 months increased MP density which was related to improved spatial working memory and problem approaching efficiency in healthy older adults [13]. Lutein supplementation has been shown to increase MP density and improve neural processing speed in young adults [14] and verbal fluency in older women [15]. In the latter study, supplementation with both lutein and docosahexaenoic acid (DHA) improved cognitive status beyond supplementation with either alone [15]. The combination resulted in additional cognitive benefits involved in spatial working memory and rate of learning. Consistent with this finding, a significant interaction has been reported between brain concentrations of lutein and DHA as a predictor of cognitive scores measuring working memory, verbal fluency, and dementia in centenarians [16]. Together, these findings indicate that lutein may function to improve several domains of cognition in interaction with DHA.

Although DHA is important for cognition [17–19], like other PUFAs, it is susceptible to oxidation due to the high number of double bonds in its structure. Nonenzymatic oxidation of DHA has been documented *in vivo* [20], including in the brain [21–23], especially in conditions of oxidative stress such as aging and neurodegenerative disease [24,25]. Therefore, it is possible that lutein functions in the brain as an antioxidant to protect DHA, and potentially other major PUFAs, such as arachidonic acid (AA), from oxidation. However, the direct relationship between lutein content and PUFA oxidation in the brain has not previously been reported.

Lutein accumulates within membranes due to its amphipathic structure [26]; however, its distribution among different types of brain membranes with distinct functions is unknown. Determining the subcellular localization of lutein is a critical first step towards understanding its potential functions in the brain. The study objective was to determine the distribution of lutein in brain membranes from regions involved in different domains of cognition, and characterize the relationship of membrane-specific lutein with DHA and AA oxidation products in the brain. Brain regions of interest included the prefrontal cortex (PFC), cerebellum (CER), striatum (ST) and hippocampus (HC). The PFC plays a key role in working memory, planning, cognitive flexibility, thought, and language [27]. CER is involved in motor control and both motor and non-motor cognitive function [28,29]. ST is involved in working memory, decision-making, rule learning, attention control, as well as motivation and perception of reward [30], and HC is a key structure for spatial memory and the formation and retrieval of long-term memories [31]. This study was performed in rhesus monkeys because they are a well-accepted model for human brain physiology and are known to absorb and store lutein in neural tissue as do humans, but not other mammalian species [32–34].

## Materials and methods

### Animal welfare: Diet and environment

Rhesus monkeys (*Macaca mulatta*, 10 female and 3 male, 7–20 years of age) were fed a standard stock diet (Monkey Diet 5037/5038, LabDiet, St. Louis, MO) at least twice a day, along with a variety of supplemental seasonal fruits and vegetables daily. The stock diet contained ~16 μmol/kg lutein, ~6 μmol/kg zeaxanthin, ~5 μmol/kg β-carotene, ~1 μmol/kg α-carotene, and ~0.1 μmol/kg cryptoxanthin, confirmed using previously reported methods [35]. The average amount of lutein consumed from the stock diet was ~2 mg total per day or 0.5 μmol/kg body weight/day, equivalent to humans consuming ~15–20 mg/day, which is an achievable dietary intake but is higher than typical consumption in the U.S. (1–3 mg/day) [1]. The n-6 PUFA content of the diet was predominantly linoleic acid (1.7% of ration) with less than 0.01% of ration being AA. The n-3 PUFA content was 0.13% of ration, mainly contributed by linolenic acid (0.10%), but also containing DHA (~0.01% of ration) (LabDiet, St. Louis, MO). Four of these monkeys, all female, were additionally orally supplemented daily with lutein at ~1 μmol/kg body weight/day (~4.5 mg/day) and zeaxanthin at ~0.1 μmol/kg body weight/day (~0.5 mg/day) for 6 months prior to termination in order to increase the range of lutein concentrations among the brain samples. This dose of lutein (equivalent to 30–40 mg/day for adults weighing 60–75 kg) is attainable from the diet [36] but is more commonly achieved through supplementation in humans. In Cynomolgus monkeys, no adverse effects were observed when oral doses of lutein up to 35 μmol/kg body weight per day were given for 52 weeks [37]. Additionally, no adverse effects of lutein have been reported in humans [38] and no tolerable upper intake levels for lutein have been established by the Institute of Medicine. The supplement, in the form of gelatin beadlets containing unesterified lutein and zeaxanthin in a starch-based matrix (DSM Nutritional Products Ltd.), was mixed into various preferred treats such as marshmallow, peanut butter, or chocolate. Characteristics of monkeys in the stock diet and lutein/zeaxanthin (L/Z) supplemented group are shown in Table 1. Age, sex, and body weight did not differ between treatment groups. All females in the study were intact (not ovariectomized) and having normal (28-day) menstrual cycles.

**Table 1:** Rhesus monkey characteristics (mean ± SEM) in stock diet and L/Z supplemented treatment groups.

Characteristics	Stock Diet (n = 9)	L/Z Supplement (n = 4)	P values
**Age**	11.7 ± 1.1	13.9 ± 2.7	0.37
**Sex (male/female)**	3/6	0/4	0.49
**Body Weight, kg**	7.87 ± 0.75	7.16 ± 0.65	0.56

Means were compared using Student’s T test. Fisher’s Exact test was used for the categorical variable (sex).

### Ethics statement

The study was in compliance with all institutional and federal regulations on the use of laboratory animals as well as the Guide for the Care and Use of Laboratory Animals [39]. Procedures were approved by the Institutional Animal Care and Use Committee (IACUC) of Oregon Health & Science University (Protocol IS00003766). Throughout the study, animals were housed at the Oregon National Primate Research Center (ONPRC), which is fully accredited by the Association for the Assessment and Accreditation of Laboratory Animal Care International. All L/Z supplemented monkeys (n = 4, female) and 4 of the 9 stock diet-fed monkeys (3 female, 1 male) were born at the ONPRC. The other stock diet-fed monkeys (3 female, 2 male) were obtained from either Shin Nippon Biomedical Laboratories, the largest contract research organization and laboratory animal breeding company in Japan (n = 4), or Valley Biosystems in West Sacramento, California (n = 1). These monkeys lived at the ONPRC for 3–4 years before their inclusion in the study.

Professional care was provided by the Oregon National Primate Research Center (ONPRC) Division of Comparative Medicine and all animals were observed at least twice a day by trained veterinary technicians. In addition to rotating dietary supplements of fruits and vegetables, animals were provided with environmental enrichments including a changing variety of toys and enrichments devices. For the unsupplemented group, brain and serum samples were made available to the study through the ONPRC Tissue Distribution Program; animals were not euthanized expressly for this study but were obtained from monkeys that were euthanized for other projects or for veterinary reasons. Euthanasia was conducted by a veterinary pathologist; animals were sedated with ketamine, and then deeply anesthetized with sodium pentobarbital according to the Guidelines of the American Veterinary Medical Association.

### Blood and brain collection

Fasting blood samples were drawn from the saphenous vein at the time of euthanasia after ketamine sedation from all supplemented animals and from 7 of the 9 unsupplemented animals. Since unsupplemented animals were not euthanized expressly for this study, blood samples were not available in every case. Blood was processed for serum (1,000 x g, 10 min, 4°C) then stored at -80°C prior to analysis. Immediately after euthanasia, PFC, CER, ST, and HC were removed from the right and left hemispheres. The regions consisted of gray and white matter, but excluded major white matter tracts. Each brain region was immediately placed on dry ice then stored at -80°C. Samples were shipped overnight on dry ice to Tufts University. For each region, the samples from the right and left hemisphere were pooled, pulverized in liquid nitrogen, aliquoted, and stored at -80°C for subsequent analyses.

### Preparation of brain membranes

Differential centrifugation with a Ficoll density gradient was performed to isolate nuclear, myelin, mitochondrial, and neuronal plasma membranes from each brain region using established, validated methods [40,41]. Briefly, pulverized brain tissue was homogenized in aqueous buffer (10 mM HEPES, 0.25 mM EDTA, 0.32 M sucrose, pH 7.2) containing protease inhibitors (cOmplete^™^ protease inhibitor cocktail, Roche) and subjected to centrifugation (1,000 x g, 4°C) to isolate the crude nuclear membrane pellet. The resulting supernatant was removed, placed in a new tube and the protocol was repeated with the remaining pellet. Supernatant from the second centrifugation was combined with the first. The combined supernatants were then centrifuged (17,000 x g, 4°C) to obtain the crude membrane pellet containing myelin, mitochondrial, and neuronal plasma membranes. The crude membrane pellet was re-homogenized in buffer without sucrose (10 mM HEPES, 0.25 mM EDTA, pH 7.2), applied to a Ficoll density gradient (consisting of 14% and 7% Ficoll solutions), and centrifuged (87,000 x g, 4°C) to separate myelin, mitochondrial, and neuronal plasma membranes. All three membranes, along with the crude nuclear membrane, were purified via centrifugation at 17,000 x g, 4°C. Pure membranes were aliquoted for carotenoid and fatty acid analyses and stored at -80°C. Membrane recovery, determined by measuring the sum of α-tocopherol levels in all membranes and supernatants and comparing to total α-tocopherol in each brain sample analyzed, was 76% ± 1%.

### Carotenoid extraction from brain regions, membranes, and serum

Extraction of carotenoids from brain regions and membranes was adapted from Park et al. [42] and has been previously described in detail [6]. Briefly, regions and membranes were homogenized in 0.3 mL saline and 0.5 mL ethanol. To the homogenate, 50 μL of echinenone (internal standard) was added with 2 mL of ethanol and the mixture was vortexed. After incubating the mixture in a 70°C water bath for 2 minutes, saponification was carried out (60°C water bath, 20 minutes) using 25% sodium ascorbate (0.5 mL) and 5% sodium hydroxide (1 mL). Samples were cooled for 5 minutes and 0.5 mL distilled water was added. Carotenoids were extracted (two times) from samples through the addition of hexane (5 mL), vortexing, and centrifugation (1,000 x g, 10 minutes, 4°C). Extracts were dried under nitrogen and resuspended in a 1:1 mixture of ethanol and methyl tert butyl ether (75 μL). Samples were centrifuged in a microfuge (Eppendorf 5415D, Eppendorf, NY) at 2000 *g* for 2 minutes to remove any precipitate. Clear supernatant was transferred to HPLC inserts and injected in a reverse-phase HPLC system with a C30 carotenoid column (3 μm, 150 x 4.6 mm, YMC America). Serum was analyzed to assess dietary carotenoid intake. Serum was also collected from all L/Z supplemented monkeys, providing a total of 11 serum samples for analysis. Carotenoids were extracted and analyzed from serum using a modified Folch method and quantified using reverse-phase HPLC [34]. The lower limit of detection was 0.2 pmol for carotenoids. Interassay coefficients of variation (CV) were 4%. Brain region and membrane data are expressed as ng/mg protein.

### Fatty acid determination in brain regions

Total lipids were extracted overnight (4°C) from brain sample homogenates using a modified Folch method [43]. The resulting total lipid fractions were saponified, methylated, and analyzed for DHA and AA using an established gas chromatography method [44]. Peaks of interest were identified by comparison with authentic fatty acid standards (Nu-Chek Prep, Inc. Elysian, MN) and expressed as a concentration (μg/mg protein). On average, the interassay CV ranges from 0.5 to 4.3% for fatty acids present at levels >5% of total fatty acids.

### Protein determination in brain regions and membranes

The resulting delipidated brain tissue/membranes from overnight total lipid extraction were digested in 1N sodium hydroxide for the determination of protein using the bicinchoninic acid (BCA) assay (Pierce Inc., Rockford, IL). Brain regions and membranes were digested for 8 and 5 days, respectively.

### PUFA oxidation determination in brain tissue

Neuroprostanes (NP) and isoprostanes (IsoP) are the collective names for a group of compounds formed from the oxidation of DHA and AA, respectively. Total NP and IsoP were extracted and quantified using published methods [45,46] with modifications. Briefly, lipids were extracted from homogenized samples of each brain region using the Folch method. The lipid extract was saponified to release esterified NP and IsoP and neutral lipids were removed from the resulting mixture using hexane. Samples were acidified to pH 3 to protonate NP and IsoP carboxylic acid groups. Addition of an internal standard, [^2^H_4_] 15-F_2t_-IsoP (Cayman Chemicals, Ann Arbor MI), was added prior to extraction with ethyl acetate. NP and IsoP were converted to pentafluorobenzyl (PFB) esters, and subjected to HPLC (Agilent 1050) to isolate NP and IsoP as PFB esters as previously described [45]. PFB ester fractions were collected, converted to trimethylsilyl ether derivatives, and quantified using GC/MS [45]. Selective ion monitoring was used at *m/z* 593 for NP, *m/z* 569 for IsoP and *m/z* 573 for the internal standard. Inter-assay CV was 10%. Analysis was completed in PFC, CER, and ST samples from all unsupplemented monkeys (n = 9) and two of the four L/Z supplemented monkeys due to instrument failure. Due to the small size of the HC, there was insufficient tissue available from this region for this analysis.

### Statistical analysis

Given the novelty of this work, no information was available on which to base sample size calculations. Carotenoid and fatty acid data are expressed as mean ± standard error of mean. For serum, total concentrations (sum of *cis* and *trans* isomers) of carotenoids were used. In the brain, only the *trans* isomers of carotenoids were detected. Although monkeys analyzed in this study spanned an age range of 13 years, all monkeys were of adult age. Additionally, age did not significantly differ between stock diet and L/Z supplemented monkeys, allowing for comparisons between treatment groups. Two-tailed Student’s T test was performed to determine differences in serum and brain region concentration for each carotenoid between stock diet-fed and L/Z supplemented monkeys. One-way analysis of variance (ANOVA) with Tukey’s HSD was performed to determine differences in carotenoid concentration in serum as well as across brain regions. Due to significant differences in carotenoid concentrations across the brain (P<0.05), brain regions were analyzed separately and a one-way ANOVA with Tukey’s HSD was performed to determine the subcellular distribution of lutein within each region. Pearson correlations were performed to assess the relationship between PUFA oxidation products in each region and membrane-specific lutein concentrations. For this analysis, stock diet-fed and L/Z supplemented monkeys were combined and adjusted for treatment (stock diet vs supplement). Furthermore, aging is associated with increased oxidative stress [47]. Given the age range of monkeys (7–20 years old), we considered age as a covariate. Using partial correlations allows for a precise, easily interpretable estimate of the association between lutein concentration and PUFA oxidation independent of age as well as treatment effects. For all analyses, significance was set at the 0.05 level and performed using SAS 9.4.

## Results

### Serum carotenoid profile in adult rhesus macaque

**Fig 1:**
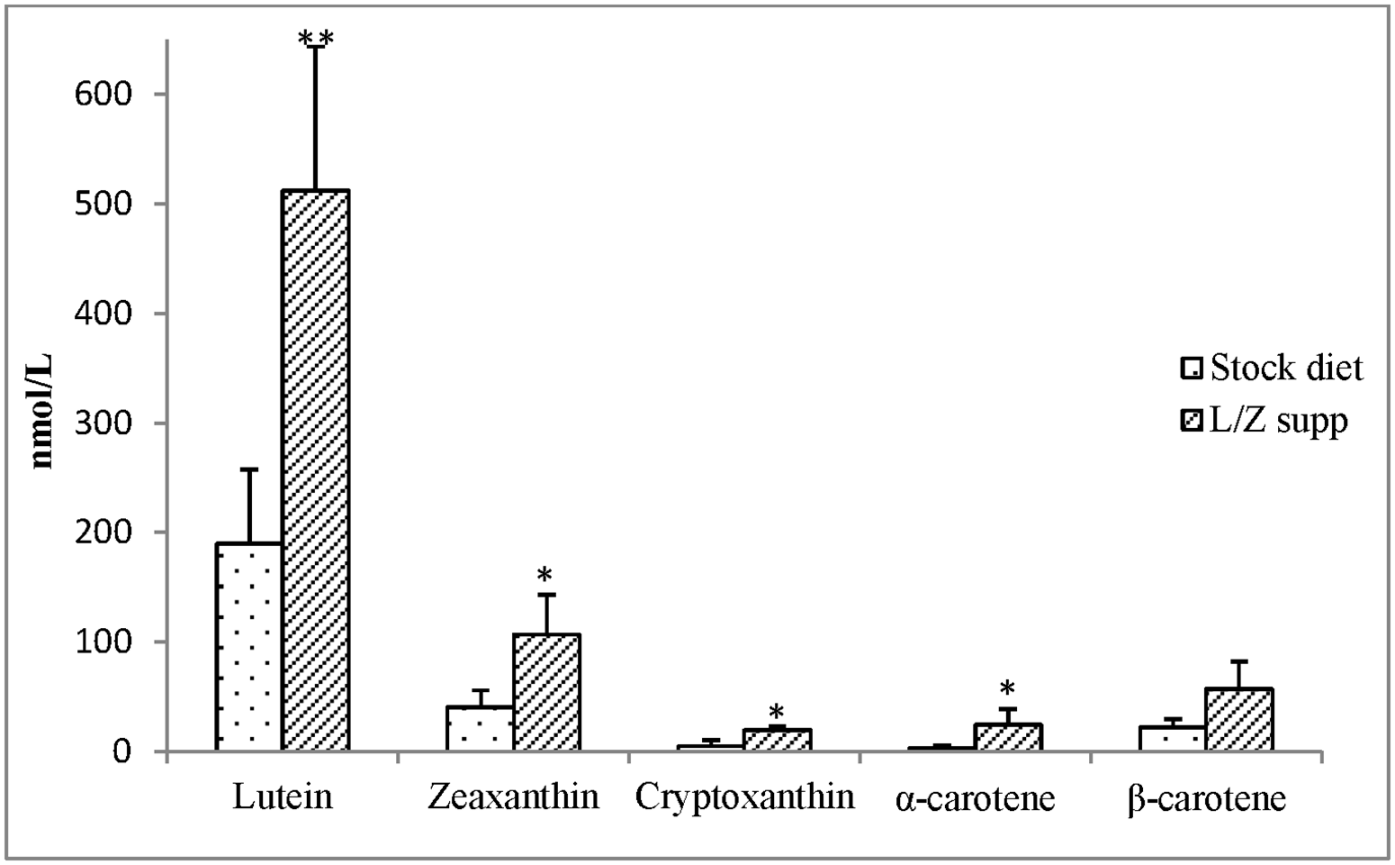
Serum carotenoid profile (nmol/L, mean ± SEM) in stock diet-fed (n = 7) and lutein/zeaxanthin (L/Z) supplemented (4.5 mg & 0.5 mg per day, respectively) adult rhesus macaques (n = 4). Difference in serum carotenoid concentrations in L/Z supplemented vs stock diet-fed monkeys (Student’s T-test) **P<0.05; *P<0.1.

Serum carotenoid profiles (*cis* + *trans* isomers) in stock diet-fed versus L/Z supplemented monkeys are presented in Fig 1. Lutein concentrations were significantly higher in L/Z supplemented (513 ± 131 nmol/L) compared to stock diet-fed monkeys (190 ± 68 nmol/L) (P<0.05). Compared to the stock diet-fed monkeys, L/Z supplemented animals also tended to have higher levels of serum zeaxanthin (P<0.08). Serum cryptoxanthin (P = 0.09), α-carotene (P = 0.08), and β-carotene (P = 0.1) also tended to be higher in L/Z supplemented monkeys compared to those fed the stock diet only. Given that the supplement did not contain these carotenoids, the trend for an increase in serum concentrations of these carotenoids may be due to a “sparing effect” against oxidation from the L/Z supplements. Such a sparing effect has been previously observed due to supplementation with β-carotene [48,49]. Lycopene was not present in the diet, and thus it was not detected in serum.

### Carotenoid concentrations in different regions of the brain

#### Stock diet-fed monkeys

**Fig 2:**
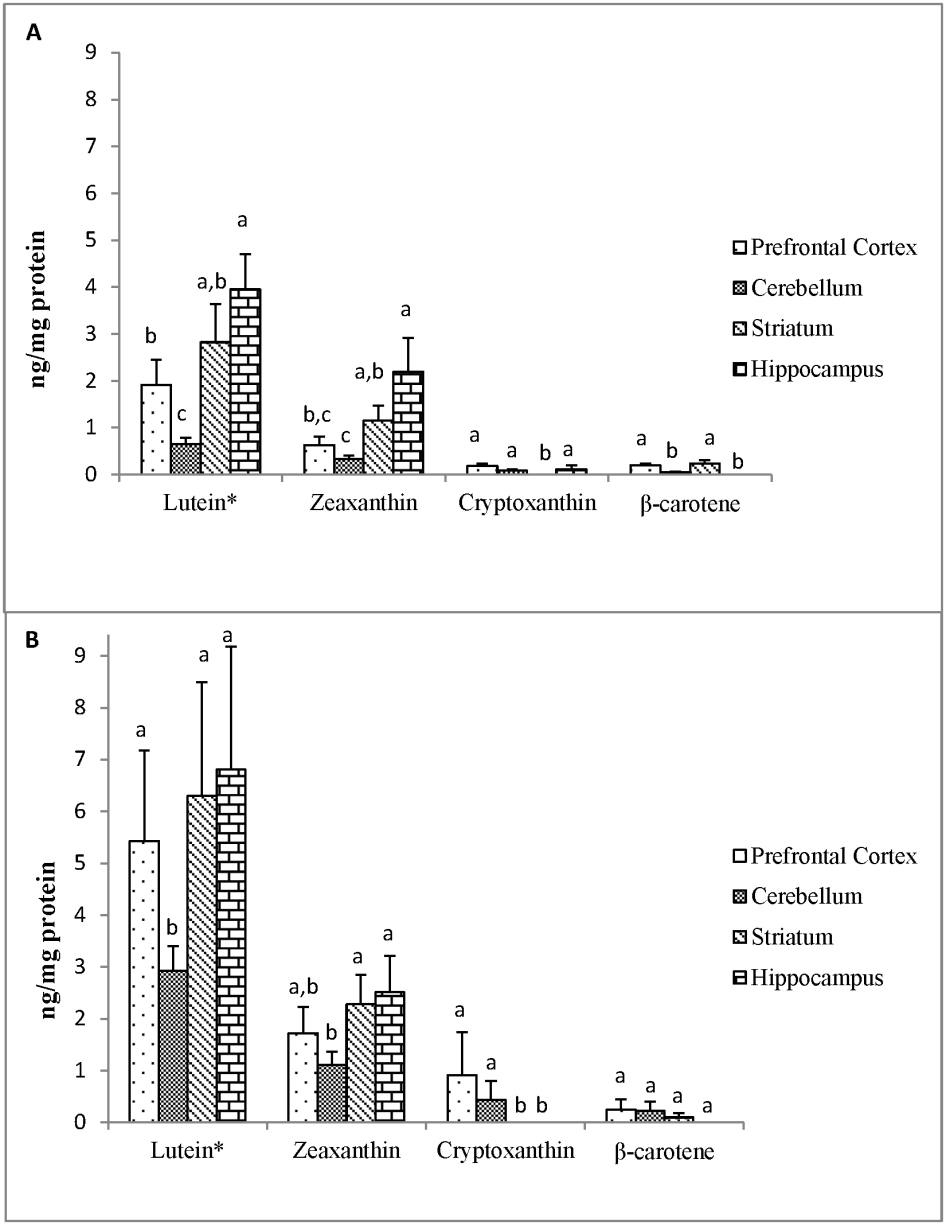
Mean (±SEM) carotenoid concentrations (ng/mg protein) in different regions of the brain from (A) stock diet-fed (n = 9) and (B) lutein/zeaxanthin (L/Z) supplemented (n = 4) adult rhesus macaques. *Lutein was significantly greater than all other carotenoids within each brain region. Bars with different superscripts across brain regions for each individual carotenoid are significantly different according to Tukey’s HSD test (P<0.05).

The carotenoid content in PFC, CER, ST, and HC of stock diet-fed monkeys are presented in Fig 2A. Only the *trans* isomers of carotenoid were detected in brain regions. Similar to serum, lutein was the major carotenoid detected in the brain and had significantly greater concentrations than all other carotenoids within each region in both stock diet-fed and L/Z supplemented monkeys (P<0.05). Comparing carotenoids across brain regions in stock diet-fed monkeys, lutein concentration was significantly lower in CER compared to all other regions (P<0.05). Lutein concentrations in HC were significantly higher than PFC (P<0.05), and ST had concentrations of lutein that were not different from HC and PFC. Zeaxanthin followed a similar distribution pattern among regions. Cryptoxanthin concentrations were similar across PFC, CER, and HC, but not detected in ST. β-Carotene concentrations were higher in PFC and ST compared to CER, but not detected in HC (P<0.05). Lutein concentrations did not differ between male and female stock diet-fed monkeys (S1 Fig).

#### L/Z supplemented monkeys

As in stock diet-fed animals, lutein concentrations were significantly lower in CER compared to all other regions in L/Z supplemented monkeys (P<0.05) (Fig 2B). However, lutein concentrations in PFC, ST, and HC were not significantly different. Zeaxanthin concentrations in CER were significantly lower than in STR and HC (P<0.05), but not PFC. Cryptoxanthin was not detected in ST or HC, and β-carotene was not detected in HC. In PFC and CER, lutein concentrations were significantly greater in L/Z supplemented monkeys compared to monkeys fed stock diet alone (P<0.05). Lutein tended to be higher in ST of L/Z supplemented monkeys compared to stock diet-fed monkeys, but this difference was not statistically significant (P = 0.09). HC lutein concentrations did not differ between the two groups.

#### Relationship between serum and brain lutein

Matched serum (*cis* + *trans*) and brain (*trans*) lutein concentrations were strongly and positively correlated for all regions, adjusted for age (P≤0.01, Table 2). Correlations between serum *trans*-lutein and brain lutein content were significant across PFC, CER, ST, and HC, and were stronger than the respective associations between serum *cis*-lutein and brain lutein content for all regions, especially the HC, which was the only region where lutein content was not significantly associated with serum *cis*-lutein (r = 0.58, P = 0.08).

**Table 2:** Relationship between matched serum lutein (nmol/L) and brain lutein (ng/mg protein) content in prefrontal cortex (PFC), cerebellum (CER), striatum (ST), and hippocampus (HC) in adult rhesus macaques (n = 11*).

		PFC	CER	ST	HC
**Serum**	**Total (*cis + trans*)**	0.87 (p = 0.001)	0.83 (p = 0.003)	0.91 (p = 0.0002)	0.74 (p = 0.01)
	***Trans* only**	0.89 (p = 0.0006)	0.84 (p = 0.002)	0.93 (p<0.0001)	0.78 (p = 0.008)
	***Cis* only**	0.74 (p = 0.01)	0.78 (p = 0.007)	0.76 (p = 0.01)	0.58 (p = 0.08)

*Serum not available for two monkeys. Values are Pearson’s correlation coefficients (r).

### Distribution of lutein in subcellular membranes of brain regions

**Fig 3:**
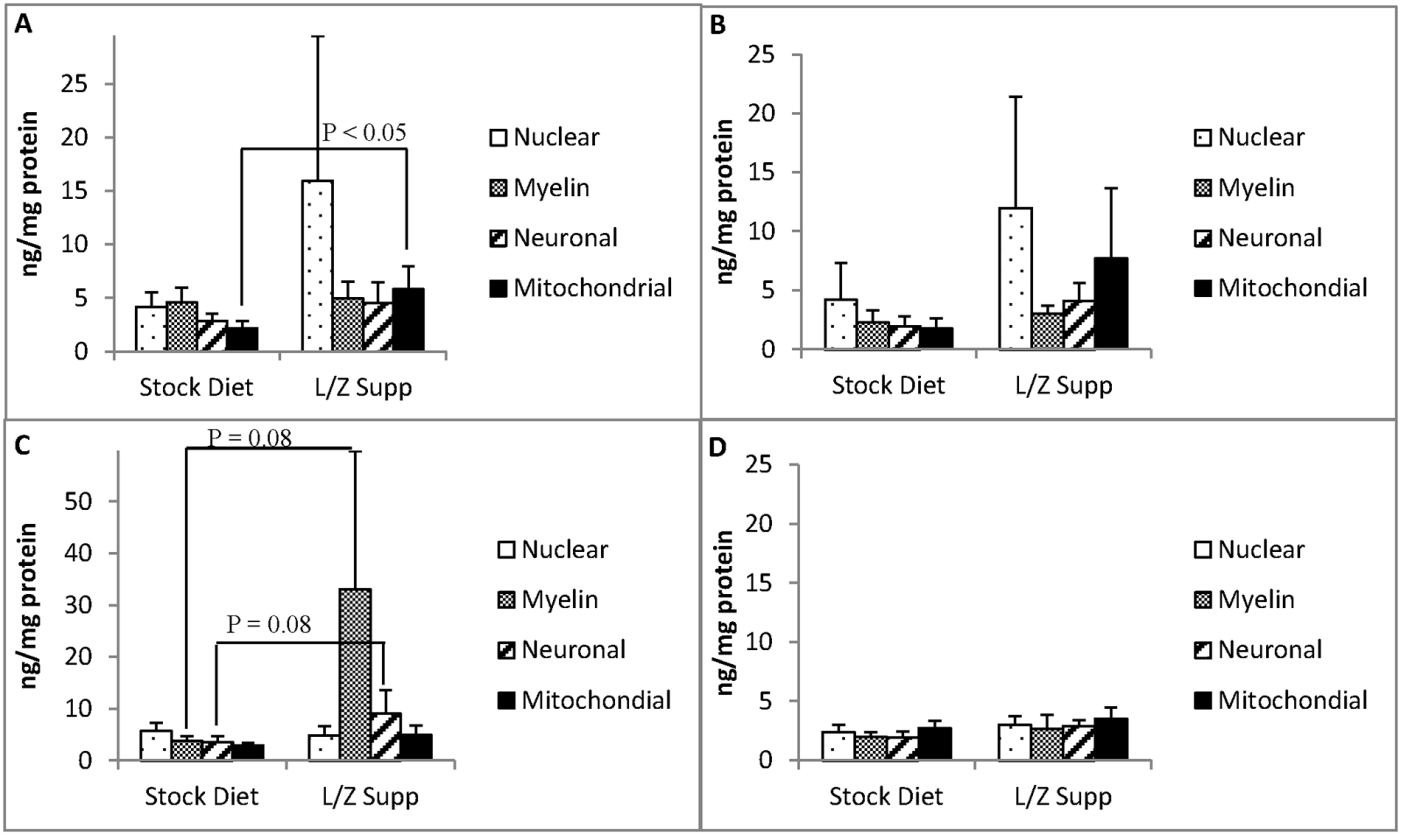
Membrane lutein concentrations (ng/mg protein, mean ± SEM) in (A) prefrontal cortex (B) cerebellum (C) striatum (D) hippocampus of adult rhesus macaques fed stock diet (n = 9) or lutein/zeaxanthin (L/Z) supplement (n = 4). P<0.05 indicates significant difference according to Tukey’s HSD test.

The range of mean membrane carotenoid concentrations among brain regions in both stock diet-fed and L/Z supplemented monkeys are reported in S1 Table. Lutein was the predominant carotenoid detected in each subcellular membrane type among brain regions. In addition, it was the only carotenoid detected in every membrane analyzed (208 total). The membrane distribution of lutein (ng/mg protein) in PFC, CER, ST, and HC, respectively, in stock diet-fed and L/Z supplemented monkeys is presented in Fig 3A–3D.

#### Stock diet-fed monkeys

In monkeys fed stock diet alone, there were no statistically significant differences in lutein concentration across membranes types within each region. However, in PFC, myelin lutein tended to be greater than lutein in mitochondrial membranes (Fig 3A, P = 0.09). In ST, lutein in the nuclear membrane tended to be greater than that in mitochondrial membranes (Fig 3C, P = 0.06). Membrane lutein concentrations did not differ between male and female stock diet-fed monkeys (S2 Table).

#### L/Z supplemented monkeys

Membrane lutein concentrations were highly variable among L/Z supplemented monkeys. No significant differences were observed in lutein concentration across membranes types within each brain region. Lutein concentrations in PFC mitochondrial membranes were significantly greater in L/Z supplemented animals compared to monkeys fed stock diet only (Fig 3A, P<0.05). The mean lutein concentrations in ST myelin and neuronal plasma membranes of L/Z supplemented monkeys were 8.5 and 2.5 times greater than the respective membrane levels in stock-fed monkeys; however, these differences did not reach statistical significance (Fig 3C, P = 0.08).

### Relationship between membrane lutein and PUFA oxidation in brain regions

Mitochondrial membrane lutein levels were inversely associated with DHA oxidation products (NP) in both PFC and ST (P<0.05) (Table 3, S2 Fig). ST myelin lutein concentration, and total ST lutein concentrations, tended to be inversely associated with DHA oxidation (P = 0.08 for both). Membrane lutein was not significantly correlated with AA oxidation products (IsoP); however, total lutein concentration in PFC tended to be inversely associated with AA oxidation (P = 0.08) (Table 3, S3 Fig).

**Table 3:** Partial correlations between membrane lutein^#^ and neuroprostanes and isoprostanes from different brain regions of rhesus macaques (n = 11^).

	Total	Nuclear	Myelin	Neuronal	Mitochondrial
	Neuroprostanes				
**Prefrontal Cortex**	-0.48	--	-0.48	--	-0.69**
**Cerebellum**	--	--	--	--	-0.48
**Striatum**	-0.59*	--	-0.59*	-0.48	-0.68**
	Isoprostanes				
**Prefrontal Cortex**	-0.60*	--	--	-0.51	-0.53
**Cerebellum**	--	--	--	--	--
**Striatum**	--	--	--	--	--

^#^ log transformed;

^Analysis was not performed for two of the L/Z supplemented monkeys;

**P<0.05,

*P<0.1

Partial correlations adjusted for age and treatment (stock diet vs. stock diet + supplement)

Weak correlation: r = 0.3–0.5; Moderate correlation: r = 0.50–0.70; Strong correlation: r = 0.70–0.99 [50].

**Fig 4:**
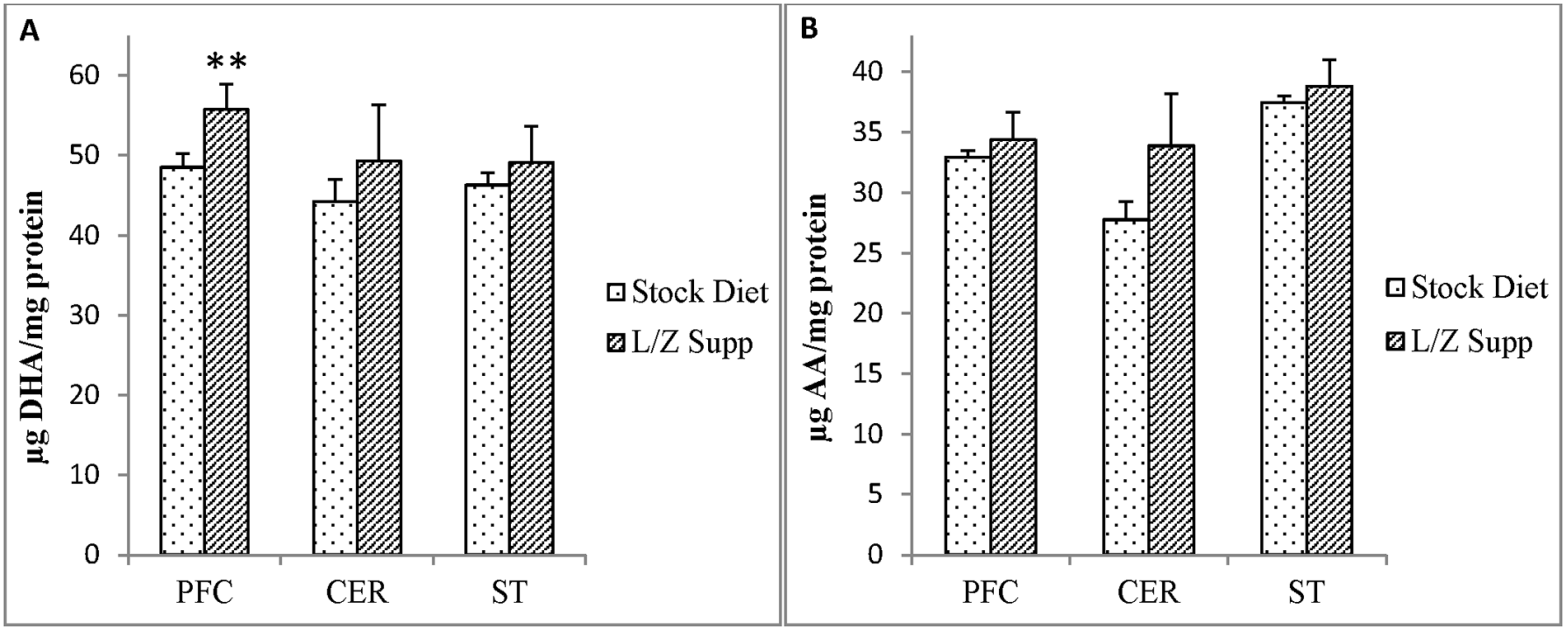
Mean concentrations (μg/mg protein) among brain regions (prefrontal cortex, cerebellum, striatum) of (A) docosahexaenoic acid (DHA) in stock diet (n = 9) and L/Z supplemented rhesus monkeys (n = 4) and (B) arachidonic acid (AA) in stock diet (n = 9) and L/Z supplemented rhesus monkeys (n = 4). Difference in concentrations in L/Z supplemented vs stock diet-fed monkeys (Student’s T-test) **P<0.05.

Given the observed associations between membrane lutein content and PUFA oxidation in brain regions, we compared brain DHA and AA concentrations in stock diet-fed and L/Z supplemented monkeys (Fig 4). DHA concentrations were significantly higher in L/Z supplemented monkeys (n = 4) compared to stock diet-fed monkeys (n = 9) in PFC (P<0.05), but not CER and ST (Fig 4A). AA concentrations were not significantly different between treatment groups for any brain region (Fig 4B).

## Discussion

This study is the first to report on the membrane distribution of lutein in the primate brain and to examine the relationship between membrane lutein concentrations and brain PUFA oxidation products. A number of novel findings were elucidated from these analyses. Firstly, *all trans* lutein was the only dietary carotenoid found in all brain regions/membranes. Secondly, further supplementation of lutein to a stock diet led to higher concentrations of lutein in the brain. Thirdly, lutein did not preferentially accumulate in one specific membrane type and was highly variable among monkeys, indicating that a number of underlying factors (e.g. genetics, lutein transport, dietary intake) dictate deposition of lutein into brain membranes. Finally, NP oxidation products were inversely associated with lutein content in mitochondrial membranes, particularly in PFC, which was also the only region and membrane type that was significantly enriched with L/Z supplementation. The PFC was also the only region to have higher DHA content with L/Z supplementation. This suggests lutein accumulating in mitochondrial membranes may be associated with an important antioxidant role in the brain, particularly the PFC.

### Regional distribution of lutein in rhesus monkey brain

Consistent with previous studies in humans and non-human primates [5–8,51], lutein was the predominant carotenoid found in all brain regions tested. In our study, this result is partially due to dietary intake, since the stock diet contained higher amounts of lutein than all other carotenoids. However, human studies in both adults [8,52] and infants [7] have demonstrated a preferential uptake of lutein into the brain. This may be specific to its isomeric form as *cis*-lutein was not detected in any brain region, and therefore the association between serum and brain lutein content was driven solely by the *trans* isomer. Collectively, these observations indicate that uptake of the *trans* isomer of lutein into brain tissue may be more efficient than *cis*-lutein uptake. Although the *cis* isomer has been previously detected in human brain, relative to *trans* levels the amounts were extremely low and inconsistent [6,8].

Brain regions analyzed for carotenoids vary across published studies in rhesus monkeys and humans. However, there is some overlap. Brain lutein concentrations reported here are similar to those measured in a previous study that also utilized adult rhesus monkeys and reported differences in lutein concentration among brain regions [6]. Specifically, lutein levels in CER were lower compared to other regions, including frontal cortex (FC), occipital cortex (OC), and pons [6].

A recent study investigating brain concentrations of lutein in infant rhesus monkeys given a formula with low carotenoid concentrations after an initial period of breast feeding reported lower concentrations of lutein in ST and HC compared to the stock diet-fed adult monkeys in our study [51]. From this same study, infant rhesus monkeys given a lutein-supplemented formula for 4 months had comparable but slightly lower brain concentrations of lutein than concentrations reported in PFC, CER, ST, and HC of L/Z supplemented adult rhesus monkeys from the present study. This may be due to both a shorter duration in supplementation in the infant monkeys and shorter lifetime to accumulate L/Z in the brain compared to adult monkeys. A study in human infants determined that lutein concentrations did not differ among brain regions (including HC, PFC, FC, OC, and auditory cortex) [7].

Our findings of higher accumulation of lutein in ST, HC, and PFC (regions important for working memory, learning, and attention) compared to CER lend support to cognition studies that have reported improvements in these domains of cognitive function with dietary and supplemental lutein [8,10,13–15]. Significantly higher levels of lutein in brain regions of L/Z supplemented monkeys compared to exclusively stock diet-fed monkeys provides evidence that lutein supplementation increases brain lutein. Our data also suggest that lutein supplementation is particularly effective in enhancing lutein in brain regions with relatively lower concentration (PFC and CER) compared to regions with higher concentrations (ST and HC).

### Membrane distribution of lutein in rhesus monkey brain and its relationship to brain PUFA oxidation

In contrast to the other dietary carotenoids, lutein was detected in all brain regions and all membrane types, indicating that lutein may play an important role in the brain. With regard to its distribution among different membrane types, lutein was not observed to preferentially accumulate in any one particular membrane, concentrations were highly variable among monkeys, and lutein levels were not consistently higher in L/Z supplemented monkeys compared to stock diet-fed monkeys. It is likely that numerous factors (i.e. age and genetics) are involved in dictating the subcellular distribution of lutein within brain regions. However, similarity in membrane lutein concentrations between male and female stock diet-fed monkeys suggests that sex may not be one of these contributing factors. Previous studies investigating the role of genetics in accumulation of lutein in the retina, as measured by MP density, have determined that MP density is influenced by a number of gene variants related to carotenoid transport, uptake, and metabolism, as well as omega-3 PUFA status, and cholesterol transport and uptake [53,54]. Given evidence that lutein may be taken up into the retina and brain through similar mechanisms [5,6], studies investigating the role of these genes in relation to the high variability in membrane lutein content observed in the present study are warranted. Another factor underlying variability of brain lutein accumulation in response to intake may be differences in lutein transport in the circulation. Serum lutein is carried exclusively on lipoproteins, predominantly on high density lipoprotein (HDL), with lower amounts on low-density lipoprotein (LDL) and very-low density lipoprotein (VLDL) [55]. Emerging evidence indicates that lutein carried on HDL is specifically targeted to neural tissue (retina and brain) [56,57]. Therefore, variability in HDL levels may influence the amount of lutein deposited in the brain. Future studies investigating the effect of circulating HDL levels on brain lutein content in rhesus monkeys are needed.

Our results showed that L/Z supplemented monkeys tended to have higher myelin and neuronal lutein in ST, but not other brain regions, compared to stock diet monkeys, indicating that the magnitude of membrane lutein enrichment is likely brain region-specific. This may be due to region-specific differences in stability, transport, or binding by lutein-specific binding proteins, such as StARD3, in brain tissue [58]. It may also indicate that membrane lutein enrichment is dependent on brain region-specific needs or requirements. For example, the PFC is thought to be particularly susceptible to oxidative damage and inflammation that can impair its functions [59–61], and PFC mitochondrial membranes were the only membranes to have significantly higher levels of lutein in L/Z supplemented monkeys compared to stock diet-fed animals. Given that mitochondrial membranes are particularly prone to damage from reactive oxygen species produced in this organelle, this result suggests that accumulation of lutein in these membranes may be an important antioxidant function of lutein that is most critical for the PFC.

Our findings from the NP and IsoP analyses are consistent with this hypothesis that lutein accumulating in mitochondrial membranes may have important antioxidant implications for the PFC and also suggests that lutein may be associated with protecting DHA, but not AA, from oxidation. The inverse relationship between lutein concentration and NP was strongest in PFC and ST, and DHA concentrations were significantly higher in the PFC of L/Z supplemented monkeys compared to the stock diet-fed monkeys. This pattern of results suggests that enrichment of lutein in PFC may preserve DHA concentrations in this region and may contribute to inhibiting its oxidation by accumulating in mitochondrial membranes and reducing oxidative stress. Our findings build on previous evidence of an interaction between lutein and DHA that has been reported in the centenarian brain [16] as well as in a lutein and DHA intervention study in older women that measured changes in cognitive performance [15]. Protection of DHA from damage in the brain is likely critical for proper cognitive functioning, since this PUFA imparts anti-inflammatory effects [62], increases membrane fluidity, and participates in neuronal cell signaling [63], all of which promote brain health. However, future analyses investigating whether lutein co-localizes with DHA in brain membranes is needed before conclusions can be drawn regarding whether lutein physically and directly protects DHA from oxidation in PFC membranes.

A limitation of this study is the small sample size. Given the high variability in membrane lutein concentrations among monkeys and wide age range of the animals, it is possible that this study was underpowered for determining differences in lutein concentration among membrane types and between treatment groups. Another limitation of this correlational analysis is that the results do not provide direct support of an effect of lutein on protection of PUFA oxidation in brain tissue. However, this study is the first to elucidate the subcellular distribution of lutein in the brain and its association with brain DHA oxidation, providing guidance and rationale for future studies investigating the functions of lutein in the brain, particularly as it pertains to DHA oxidation. Lastly, NP and IsoP concentrations were determined in aliquots of whole tissue, rather than for each membrane, as it is currently not feasible to determine membrane-specific NP and IsoP concentrations due to limitations in methodology.

In conclusion, our novel findings extend the existing literature by revealing that lutein was found in each membrane sample analyzed despite wide variation in concentration among monkeys. In addition, our supplementation results are consistent with the possibility of differential membrane lutein kinetics. This study is the first to determine the membrane distribution of lutein in different regions of the primate brain and the first to demonstrate an association between membrane mitochondrial lutein content and DHA oxidation. Thus, lutein bioaccumulation in mitochondrial membranes may be associated with antioxidant benefits in the brain that protect DHA from oxidation in PFC and ST. These results provide intriguing evidence for a potential antioxidant-associated function of lutein in the brain and may direct future studies investigating the role of lutein in cognitive function.

## Supporting information

S1 Fig Mean lutein concentrations (ng/mg protein) among brain regions (prefrontal cortex, cerebellum, striatum, hippocampus) in stock diet-fed rhesus monkeys, stratified by sex (n = 3 male; n = 6 female). (DOCX)

S2 Fig Partial correlations between (A) total, (B) myelin, (C) neuronal, and (D) mitochondrial lutein concentrations and DHA oxidation products in prefrontal cortex (left panel), cerebellum (middle panel), striatum (right panel) in adult rhesus monkeys (n = 11). Partial correlations adjusted for age and treatment (stock diet vs L/Z supplement).
(DOCX)

S3 Fig Partial correlations between (A) total, (B) neuronal, and (C) mitochondrial lutein concentrations and AA oxidation products in prefrontal cortex (left panel), cerebellum (middle panel), striatum (right panel) in adult rhesus monkeys (n = 11). Partial correlations adjusted for age and treatment (stock diet vs L/Z supplement).
(DOCX)

S1 Table Range of mean membrane carotenoid concentrations (ng/mg protein) among brain regions (prefrontal cortex, cerebellum, striatum, hippocampus) in stock diet-fed (n = 9) and L/Z supplemented (n = 4) adult rhesus monkeys. (DOCX)

S2 Table Mean membrane lutein concentrations (ng/mg protein) among brain regions (prefrontal cortex, cerebellum, striatum, hippocampus) in stock diet-fed rhesus monkeys, stratified by sex (n = 3 male; n = 6 female). (DOCX)
