# Supplementary material for: Lutein accumulates in subcellular membranes of brain regions in adult rhesus macaques: Relationship to DHA oxidation products
Source: PLoS One. 2017 Oct 19;12(10):e0186767. doi: 10.1371/journal.pone.0186767 (PMC5648219; doi:10.1371/journal.pone.0186767)
Supplement: S3 Fig — Partial correlations adjusted for age and treatment (stock diet vs L/Z supplement). (DOCX) [file pone.0186767.s003.docx]

**B**

**A**

**C**
