# Supplementary material for: Lutein accumulates in subcellular membranes of brain regions in adult rhesus macaques: Relationship to DHA oxidation products
Source: PLoS One. 2017 Oct 19;12(10):e0186767. doi: 10.1371/journal.pone.0186767 (PMC5648219; doi:10.1371/journal.pone.0186767)
Supplement: S1 Table — (DOCX) [file pone.0186767.s004.docx]

**Table S1. Range of mean membrane carotenoid concentrations (ng/mg protein) among brain regions (prefrontal cortex, cerebellum, striatum, hippocampus) in stock diet fed (n=9) and L/Z supplemented (n=4) adult rhesus macaques.**

|  | Nuclear | | Myelin | | Neuronal | | Mitochondrial | |
| --- | --- | --- | --- | --- | --- | --- | --- | --- |
|  | Stock Diet | L/Z Supplemented | Stock Diet | L/Z Supplemented | Stock Diet | L/Z Supplemented | Stock Diet | L/Z Supplemented |
| Lutein | 2.4-5.7 | 3.0-15.9 | 2.0-4.6 | 2.7-33.1 | 2.0-3.6 | 2.9-9.1 | 1.8-2.7 | 3.5-7.7 |
| Zeaxanthin | 0.8-2.8 | 1.1-12.3 | 0.8-2.9 | 1.05-3.1 | 0.7-1.7 | 0.8-1.7 | 0.2-1.2 | 0.5-2.2 |
| Cryptoxanthin | 0.0-0.2 | 0.0-0.5 | 0.0-0.2 | 0.0-0.1 | 0.0-0.2 | 0.0-0.2 | 0.0-0.1 | 0.0-0.2 |
| β-carotene | 0.0-0.6 | 0.0-1.3 | 0.0-0.6 | 0.0-0.4 | 0.0-1.5 | 0.0-0.5 | 0.0-0.2 | 0.0-0.4 |
