# Supplementary material for: Lutein accumulates in subcellular membranes of brain regions in adult rhesus macaques: Relationship to DHA oxidation products
Source: PLoS One. 2017 Oct 19;12(10):e0186767. doi: 10.1371/journal.pone.0186767 (PMC5648219; doi:10.1371/journal.pone.0186767)
Supplement: S2 Table — (DOCX) [file pone.0186767.s005.docx]

|  | Nuclear | | Myelin | | | Neuronal | | Mitochondrial | |
| --- | --- | --- | --- | --- | --- | --- | --- | --- | --- |
|  | Male | Female | | Male | Female | Male | Female | Male | Female |
| PFC | 2.22 ± 1.28 | 5.16 ± 1.83 | | 6.18 ± 2.84 | 3.81 ± 1.62 | 2.10 ± 1.47 | 3.22 ± 0.77 | 1.68 ± 0.86 | 2.43 ± 0.98 |
| CER | 1.29 ± 0.77 | 5.68 ± 4.66 | | 1.79 ± 1.25 | 2.60 ± 1.35 | 3.05 ± 2.49 | 1.42 ± 0.51 | 3.07 ± 2.64 | 1.11 ± 0.23 |
| ST | 7.22 ± 1.86 | 5.33 ± 1.82 | | 8.03 ± 4.34 | 3.84 ± 0.94 | 2.71 ± 0.98 | 4.16 ± 1.39 | 3.02 ± 1.03 | 3.14 ± 0.67 |
| HC | 2.37 ± 1.40 | 2.44 ± 0.65 | | 1.61 ± 0.80 | 2.18 ± 0.48 | 1.92 ± 1.21 | 1.97 ± 0.45 | 2.82 ± 1.75 | 2.66 ± 0.57 |
