# Supplementary material for: Lutein accumulates in subcellular membranes of brain regions in adult rhesus macaques: Relationship to DHA oxidation products
Source: PLoS One. 2017 Oct 19;12(10):e0186767. doi: 10.1371/journal.pone.0186767 (PMC5648219; doi:10.1371/journal.pone.0186767)
Supplement: Supplementary file 4 [file pone.0186767.s001.docx]

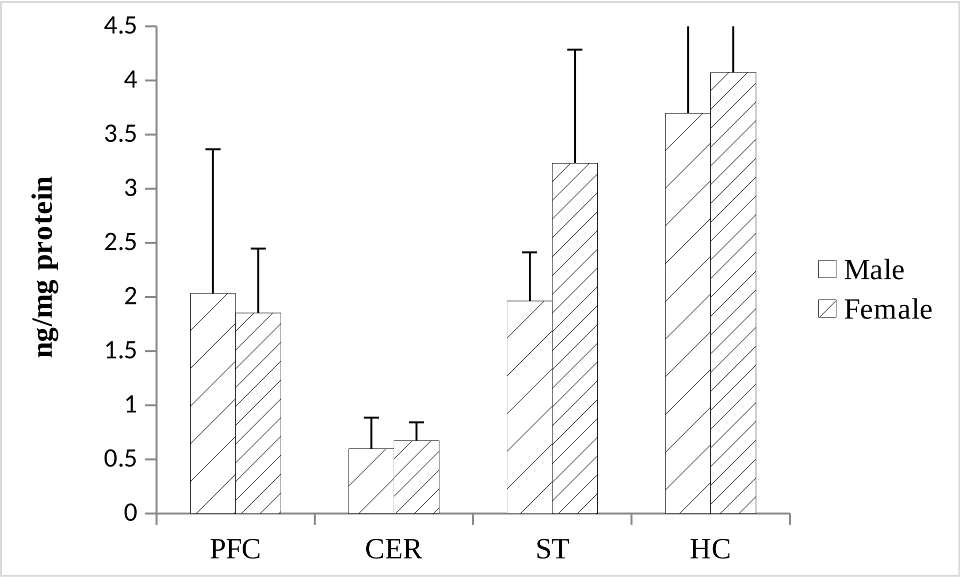

ng/mg protein
4.5
4
3.5
3
2.5
2
1.5
1
0.5
0
PFC
CER
ST
HC
Male
Female
